# From Social Stress and Isolation to Autonomic Nervous System Dysregulation in Suicidal Behavior

**DOI:** 10.1007/s11920-024-01503-6

**Published:** 2024-05-08

**Authors:** Adrián Alacreu-Crespo, Emma Sebti, Rosa María Moret, Philippe Courtet

**Affiliations:** 1 Department of Psychology and Sociology, University of Zaragoza, C/Atarazana 4, Aragon, Teruel, 44003 Spain; 2 FondaMental Foundation, Créteil, France; 3 IGF, Univ. Montpellier, CNRS, INSERM, Montpellier, France; 4 Department of Emergency Psychiatry and Acute Care, Lapeyronie Hospital, CHU Montpellier, Montpellier, France

**Keywords:** Suicide, Autonomous nervous system, Stress, Social context

## Abstract

**Purpose of Review:**

In this narrative review we wanted to describe the relationship of autonomic nervous system activity with social environment and suicidal spectrum behaviors.

**Recent Findings:**

Patients with suicidal ideation/suicide attempt have higher sympathetic nervous system (SNS) and lower parasympathetic nervous system (PNS) activity in resting conditions and during acute stress tasks compared with patients without suicidal ideation/suicide attempt. Death by suicide and violent suicide attempt also are related to SNS hyperactivation. Similarly, a SNS/PNS imbalance has been observed in people with childhood trauma, stressful life events or feelings of loneliness and isolation. Social support seems to increase PNS control and resilience.

**Summary:**

Due to the importance of the social context and stressful life events in suicidal behavior, SNS/PNS imbalance could act as a mediator in this relationship and be a source of relevant biomarkers. Childhood trauma and stressful life events may impair the autonomic nervous system response in suicidal patients. Loneliness, isolation and social support may act as moderators in acute stress situations.

## Introduction

Suicide is one of the leading causes of death. Each year, 703,000 deaths by suicide are recorded [1]. Importantly, for each suicide, there are approximately 20 people who attempt suicide. Suicide attempt (SA) is the most important risk factor for lethal suicide in the general population [2]. However, multiple risk factors are implicated in the development of suicidal spectrum behaviors (suicidal ideation, suicide attempt, and fatal suicide). Indeed, suicidal behavior is a complex phenomenon that implicates several biological, psychological, clinical, social and environmental risk factors [3].

Within these risk factors, the implication of a dysfunctional stress response in suicidal behavior has been constantly demonstrated in the literature [4, 5]. The autonomic nervous system (ANS) is an integral part of this stress response, followed by activation of the hypothalamic–pituitary–adrenal (HPA) axis. The ANS includes the noradrenergic sympathetic nervous system (SNS) and the parasympathetic nervous system (PNS), which is modulated by acetylcholine. SNS modulation by PNS allows the establishment of an adapted response to a given situation [6]. For example, in stress situations the most adaptive response is a fast SNS activation followed by fast recovery to basal levels regulated by the PNS. Thus, a balanced and context-related SNS/PNS coordination is synonymous of better health.

Dysregulation of the HPA axis has been widely studied in suicide research [7]. Conversely, fewer studies focused on the ANS. Therefore, the aim of the present narrative review was to discuss studies that explored the relationship between the ANS and suicide and to contextualize this relationship relative to the main explanatory theories of suicide that include a social component.

## Autonomous Nervous System Biomarkers and Suicidality

One of the main objectives of research on suicidal behavior is to identify biomarkers of suicide risk. Some ANS activity markers have been associated with suicidal behavior. These include cardiovascular parameters, such as heart rate variability (HRV) and variations in skin electrical conductivity, pupillary light reflex (PLR) and salivary alpha amylase concentration (sAA). Table 1 shows an overview of all ANS-related variables studied in the context of suicidal behavior.

**Table 1:** Overview of ANS-related variables

**Variable**	**Description**	**Physiological significance**
**Cardiovascular**		
Heart rate (HR)	Mean of beats per minute (bpm)	Mixed SNS/PNS activity index; higher HR = higher SNS and lower PNS activity
R-R interval	Mean of time between R – R peaks (R-R)	Mixed SNS/PNS activity index; lower R-R interval = higher SNS and lower PNS activity, raw variable for heart rate variability (HRV)
HRV-total	Total variability frequencies, 0 – 0.40 Hz	Health generic marker, higher values indicate better health
VLF-HRV	Very low frequencies, 0.0033 – 0.04 Hz	Thermoregulation and hormonal mechanisms
LF-HRV	Low frequencies, 0.04 – 0.15 Hz	Mixed SNS/PNS activity index; baroreceptor reflex activity, mental load
HF-HRV	High frequencies, 0.15 – 0.40 Hz	PNS activity marker; higher HF = higher PNS activity, respiratory sinus arrhythmia (RSA) marker
SDRR	Standard deviation of R-R intervals	In 24-h recording equivalent to HRV-total, in shorter periods equivalent to LF-HRV
RMSSD	Root mean square of successive R-R interval differences	PNS activity marker; higher RMSSD = higher PNS activity, equivalent to HF-HRV
Pre-ejection period (PEP)	Time between left ventricle depolarization and ventricular ejection	SNS activity marker, beta-adrenergic activity in hearth; lower PEP = higher SNS activity, measured by impedance cardiography (IKG)
Cardiac output (CO)	Quantity of blood pumped by the heart per minute	Hemodynamic heart efficiency, low cardiac output is equivalent to low efficiency when SNS activity is high
**Electrodermal (EDA)**		
EDA habituation	Habituation of the skin electrical conductivity in response to a stimulus	Faster habituation indicates higher SNS noradrenergic response
EDOR	ElectroDermal Orienting Reactivity (EDOR), detects EDA hyporesponsivity and fast habituation using neutral tones as stimulus	Faster habituation indicates higher SNS noradrenergic response
**Pupillary light reflex (PLR)**		
Maximum Constriction Velocity (MCV)	Peak value velocity during the changes in pupillary diameter accommodation after a luminous flash	Lower MCV is equivalent to lower PNS activity via cholinergic response
**Salivary alpha-amylase**		
	Excretion of the enzyme alpha-amylase by the salivary glands	Increases in salivary alpha-amylase indicate activation of the noradrenergic system and higher noradrenergic response

*HRV* Heart rate variability, *SNS *Sympathetic nervous system, *PNS *Parasympathetic nervous system

### Cardiovascular Parameters and Suicidality

Cardiac activity is regulated by the sinoatrial node through the SNS and PNS. Upon occurrence of an event, particularly a stressful situation, these two systems are activated to adapt the heart physiological activity and give an adapted response. The adaptive nature of this response is linked to SNS regulation by the PNS. The heart rate (HR) has classically been used to monitor SNS and PNS activation/inhibition: higher HR implies greater sympathetic activity and lower parasympathetic activity. Besides the number of beats per minute (bpm), it is possible to assess the heart rate cyclical rhythmicity, a variable that can provide more precise information on PNS activation. On the basis of the time difference between R-peaks (R-R interval), these heart cyclic periods can be monitored at different response frequencies. This group of measurements is called heart rate variability (HRV) [8]. HRV (particularly the components most closely related to the PNS) has been associated with better executive control, greater emotional stability, and more adaptive responses to social situations [9]. Consequently, its alteration is of great importance in patients with suicidal ideation and behavior.

HRV is the most studied cardiovascular parameter in suicidology, and specifically, the high frequency activity (HF-HVR) that is a more specific marker of PNS activity. Thus, an increase in HF-HRV in response to stress indicates a better adaptation of the cardiovascular system due to good vagal regulation. A study in 1461 adults with various psychiatric disorders (excluding psychotic disorders) found that the resting HR and the Root Mean Square of Successive Difference (RMSSD; a marker of cardiac vagal activity) were significantly associated with moderate to severe suicidal risk [10]. Moreover, two retrospective studies demonstrated that higher HR was related to higher odds of completed suicide [11, 12].

Several studies assessed whether autonomic cardiovascular markers can be used to identify suicidal ideation. A study found lower resting HF-HRV, low frequency activity (LF-HRV), and standard deviation of R-R peaks (SDRR) in people with past major depressive disorder (MDD) and lifetime suicidal ideation compared with people with MDD without suicidal ideation history [13]. Similarly, in a sample of young people, HF-HRV at rest was negatively associated with lifetime suicidal ideation, independently of the depression intensity [14]. However, one study in veterans did not find any association between lifetime suicidal ideation and resting HRV [15]. The presence of current suicidal ideation in adults with depression predicted lower resting RMSSD, HF-HRV, LF-HRV and total HRV compared with a group of patients with depression but without suicidal ideation [16–18]. In patients with post-traumatic stress disorder, lower HF-HRV has been related to current suicidal ideation [19].

Similar results were also observed using laboratory tasks. For instance, in college students exposed to the Stroop task and the Cyberball task (a task of social exclusion), current suicidal ideation was negatively associated with HF-HRV [20]. Children with suicidal ideation and high parental criticism exposed to a discussion with their parents had lower HF-HRV during the discussion than those without suicidal ideation or with low parental criticism [21]. Lastly, Sheridan et al. [22] showed that cardiovascular PNS regulation might be a useful marker for identifying the mechanism of change during interventions to reduce suicide risk. Specifically, they found that in a group of adolescents hospitalized in an emergency psychiatric ward, HF-HRV increased with the decrease of suicidal ideation intensity.

Concerning SA, a previous study showed lower resting HF-HRV in women with than without history of SA, even after adjusting for history of psychiatric disorders and current depressive or anxiety symptomatology [23]. Another study showed that resting HF-HRV and RMSSD were significantly lower in the patient group (n = 52 inpatients with a recent suicide attempt, < 6 months, and various psychiatric comorbidities) than in healthy controls (n = 43) [24]. Moreover, during the Trier Social Stress Test (TSST), HRV reactivity was lower in women with SA than in control patients [25]. Similarly, a study in adolescents with history of depression found lower variations in HF-HRV and greater cardiac pre-ejection period (PEP), an indicator of SNS activation, in response to stressful and sadness-inducing stimuli (sad film clip and unsolvable puzzle task) in the group with history of suicidal ideation/suicide attempt compared with healthy controls [26].

Therefore, cardiovascular parameters are altered in resting and stress conditions in suicidal patients. Notably, HRV is lower in suicidal patients than in non-suicidal patients and healthy controls. This may reflect impairments in executive control, emotional regulation and social adaptability in suicidal patients [4]. Less is known about cardiovascular hemodynamics in suicidal patients, and more research on this topic is needed to understand the cardiovascular response efficiency in these patients.

### Electrodermal Activity and Suicidality

Electrodermal activity (EDA) is the measurement of changes in the skin electrical conductivity in response to a stimulus. The activity of the cutaneous sweat glands is controlled by the SNS. The conductance between the electrodes placed on the skin surface increases with the sweat gland activity, reflecting the SNS activity [27]. For instance, repeated exposure to a neutral acoustic stimulus initially leads to an EDA increase, then a phenomenon of habituation sets in, resulting in EDA hypoactivation (EDA hypoactivity or habituation). The speed at which the habituation phenomenon occurs is proportional to the SNS activation level [28].

EDA hypoactivity has been correlated with history of SA in patients with depression and in patients in remission of depression, suggesting that this phenomenon of habituation is a marker of suicidal behavior [29, 30]. Similarly, a meta-analysis of ten studies that included 279 patients with depression and 59 healthy controls found that EDA habituation had good sensitivity (96.6%) and specificity (92.9%) for completed suicide, and also for violent suicide attempt [31]. The degree of habituation seemed to be more important in patients with history of violent suicide attempt compared with patients with history of non-violent SA or suicidal ideation alone [32]. However, more research on this topic is needed due to the heterogeneity of the methods used to induce EDA habituation, the small sample sizes, and the lack of control for antidepressant medication, although it directly affects EDA response [33].

To avoid these limitations, a European multicentric study was performed at 15 psychiatric clinics where 1573 patients were recruited using the ElectroDermal Orienting Reactivity (EDOR) test. The EDOR test uses neutral audio tones as a stimulus and allows detecting patients with EDA hyporeactivity who have unusually fast habituation. The EDOR test (hyporeactivity) was significantly related with SA at baseline and at follow-up; however, the results showed low sensitivity (< 35.4%) for SA detection [34]. In another study, 25 patients hospitalized for suicidal ideation or SA were followed with a wearable monitor and an ecological momentary assessment using negative affect self-reports for 28 days after discharge. The results showed that EDA predicted periods of suicidal ideation and that EDA, combined with negative affect self-reports, improved the prediction of acute increases in suicidal ideation severity, compared with models that used only self-reports [35].

Thus, EDA hyporeactivity consistently discriminates patients with SA and fatal suicide. However, research on the topic is obsolete and recent studies brought less enthusiastic results. EDA results confirm the increased SNS activity at rest in suicidal patients. Future research might use emotional stimuli, which are more relevant for suicidal patients, to check SNS responsivity to emotional cues.

### Pupillary Light Reflex and Suicidality

The PLR measures changes in pupillary diameter over time [36]. Pupillary constriction is controlled by the PNS, while dilation is controlled by the SNS. The two systems are continuously in equilibrium to respond to environmental stimuli [37, 38].

In a preliminary work on 30 adults without medication, McCall et al. [39] found that the pupil Maximum Constriction Velocity (MCV) was higher in patients with depression and history of SA (n = 7) or current suicidal ideation (n = 8) than in patients with depression without suicidal ideation/history of suicide attempt (n = 6) and healthy controls (n = 9). Similarly, in 26 patients with schizophrenia and 42 patients with MDD, PLR was significantly related to lifetime SA and current suicidal ideation [40]. These results are in line with the theory that the cholinergic system is in a reinforcing interaction with the noradrenergic system, leading to a state of hyperexcitability in individuals with suicidal behavior [41, 42].

### Salivary Alpha-Amylase Concentration and Suicidality

Lastly, sAA, an indirect marker of SNS, is produced by the salivary glands and its production is regulated by the noradrenergic system [43]. This marker is used to study the response to stress. After the TSST, sAA concentration was lower in adolescents with non-suicidal self-injury (NSSI; a risk factor for suicidal behavior) compared with adolescents without NSSI [44]. Similarly, the alpha amylase stress response to the TSST was blunted in individuals at risk for suicide (i.e., first-degree relatives of suicide completers) compared with individuals without family or personal history of suicidal behavior [45]. Again, a dysfunctional stress response due to ANS activation changes is related to suicidal risk.

## Suicide, Social Context and ANS

The social context plays a significant role in suicide behavior, as outlined in the main theories of suicide. For instance, the stress-diathesis model of suicide [46] proposes that suicidal acts are the consequence of the interaction between internal stressors (relative to the person, such as a depressive disorder) or external stressors (stressful life events, such as hostile work conditions) and a diathesis for some suicide-related traits (e.g. sensitivity to social distress). As mentioned in the previous section, a dysfunctional stress response to social stressors is one of the main risk factors for suicide [4, 5]. Moreover, early life stress, such as a childhood trauma, can modify the brain networks via epigenetic pathways, thus increasing suicidal vulnerability [5].

Other theories of suicide focus on social variables that facilitate or contribute to suicidal ideation and behavior. In the interpersonal-psychological theory of suicide [47], suicidal ideation can originate from feelings of perceived burdensomeness and thwarted belongingness. The three-step theory of suicide [48] explains that the change from low suicidal ideation to moderate or strong suicidal ideation depends on perceiving a sense of connectedness. In the motivational-volitional model of suicide [49], feelings of defeat and/or humiliation and entrapment lead to suicidal ideation. Defeat and humiliation may occur due to social rejection, social exclusion for different reasons (e.g. racism or homophobia [50]) or loss. Furthermore, the transition from defeat/humiliation to entrapment is modulated by threat-to-self moderators (e.g. coping). Motivational moderators modulate the probability that entrapment might lead to suicidal ideation. For instance, it is thought that experiencing social support and connectedness buffer against suicidal ideation [51, 52].

Therefore, variables like loneliness, social isolation, stressful live events and social support are essential to understand suicidal behavior. All these social variables have an impact on ANS responsivity. The next subsections discuss the impact of social factors on the relationship between ANS and suicide risk.

### Loneliness and Social Isolation

Loneliness is defined as the subjective feeling of distress experienced when the quality or quantity of social relationships is perceived as deficient [53]. It has been associated with detrimental effects on health, such as cardiovascular and mental health problems [54]. A term close to loneliness is social isolation; however, loneliness describes a subjective feeling and social isolation an objective condition. Social isolation is defined as a deficiency in the quantity and quality of interactions with other people [55]. Fewer studies on social isolation have been published, possibly because loneliness has a greater influence on health than objective variations in social engagement. Both loneliness and social isolation can predict suicidal ideation and behavior [56, 57].

Despite its health implications, loneliness effect on the stress system remains unclear. A systematic review found that higher levels of loneliness were associated with high blood pressure at rest [58]. No relationship between loneliness and catecholamines was observed (based on two studies). However, cardiac responses were blunted (e.g. lower cardiac output and HR) in lonely individuals [59]. Moreover, Hawkley et al. [60] found that loneliness was associated with higher urinary epinephrine concentration in resting conditions. Conversely, another study did not find that HRV at rest predicted loneliness [61].

Furthermore, higher loneliness levels, measured with the UCLA Loneliness Scale, were associated with larger increases in HRV after writing about a loneliness experience [62]. Some evidence suggests that in individuals with high loneliness scores, a high level of parasympathetic activity may increase the probability to engage in approaching behaviors, thus reducing loneliness [63]. Moreover, oxytocin, a neuropeptide and hormone related to social interpersonal interactions, increases HF-HRV and decreases PEP. However, higher loneliness levels seem to hinder oxytocin beneficial effects on the ANS, diminishing the parasympathetic activity [64]. On the other hand, one study reported that delayed blood pressure recovery after a stressful task was associated with social isolation [65].

### Stressful Life Events

As stated by the stress-diathesis model of suicide, the experience of stressful life events (SLE) is a risk factor for suicidal ideation and behavior [66]. SLE are conceptualized as circumstances that involve a change in the life of an individual that may need a readjustment afterwards [67]. These include divorce or separation, getting fired, work-related stressors, grief or discrimination. SLE can activate and dysregulate the ANS response and they might affect the individual’s stress system through the effort required to maintain stability throughout these changes (i.e. allostatic load) [68]. Therefore, efforts are needed to provide better support to those who experience SLE.

Psychosocial/work-related stress has been associated with a disbalance in autonomic activity, specifically decreased parasympathetic activity [69]. Unsafe work conditions (e.g. inhalation of particles), work-related stressors (e.g. fatigue) and night shifts have been associated with low HF-HRV [70]. Additionally, self-reported stress due to a divorce can predict lower cardiac vagal control by interacting with a polymorphism in the serotonin transporter gene [71]. Moreover, high HF-HRV in recently divorced adults was positively associated with the divorce-related stress and blood pressure reactivity during the follow-up [72].

Grief is another well-known life stressor. This can concern the death of a spouse or of a close family member. Indeed, such events are ranked as among the most stressful by the Social Readjustment Rating Scale [73]. O’Connor et al. [74], found that HR was higher in participants who lost a loved one than in participants with depression and controls. However, they did not find any difference in HRV among groups. Conversely, Fagundes et al. [75], found that HRV was lower in newly widowed individuals than controls. The effect of grief on autonomic activity is also influenced by the time since the loss. For instance, grief was associated with higher HR and lower HRV at week 2 than month 6 post-loss [76]. Finally, grief severity also might influence the baseline EDA and could explain the reduced HF-HRV reactivity to some emotional stimuli [77].

Discrimination is another important type of life stress. In their review, Panza et al. [78] found evidence indicating that racism may increase blood pressure. Moreover, in all included studies (n = 5), racism was associated with a decrease in HRV. Racism-related stress has also been related to increased EDA [79]. Similarly, heart rate in response to the TSST was higher in gay/bisexual men than in straight men, and blood pressure in response to the same stressor was marginally higher in lesbian/bisexual women than straight women. No difference was observed when accounting for the sexual orientation disclosure [80].

### Childhood Trauma, Neglect and Early Losses

Childhood maltreatment is a risk factor for suicidal behavior [81]. Childhood maltreatment is defined as any form of violence perpetrated against a minor. It can be divided into physical abuse, emotional abuse, sexual abuse, physical neglect, and emotional neglect [82].

In a recent meta-analysis [83], childhood maltreatment did not seem to alter resting vagal activity. However, when accounting for age and psychopathology, HRV at rest was decreased in people who reported childhood maltreatment. The meta-analysis by Wesarg et al. [84] explored the relationship between childhood adversity and resting vagal activity and reactivity. Childhood adversity is a broader concept than childhood maltreatment and includes, among others, childhood maltreatment, poverty, death of a parent, witnessing a traumatic event. Childhood adversity was not associated with vagal activity or reactivity. However, when considering the psychiatric diagnosis or direct adversities (e.g. maltreatment) as moderators, a relationship was observed between childhood adversity and reduced resting vagal activity. Moreover, using a laboratory stress, childhood adversity was associated with lower reactivity when the adversity was experienced more distantly in the past.

On the basis of the findings of both meta-analyses, the current knowledge on the topic suggests that vagal functioning is altered only in individuals who experienced specific subtypes of child adversity and among specific subgroups of individuals.

### Social Support

Social support is a protective factor for lifetime SA [51]. The stress-buffering hypothesis proposes that social support may shield the individual from the detrimental effects of stress [85]. This was confirmed by a meta-analysis showing that experiencing social support was related to health and longevity (n = 1,458 million) [86]. The perception of social support might affect the ANS, thus inducing (sympathetic dominance) or inhibiting (parasympathetic dominance) threat responses, depending on the support availability [86]. Measures of social support can be divided into structural (existence or not of social networks) and functional (whether support is perceived as available and actual support is received/provided) [85, 87].

One of the most common *structural measures* is civil status. For instance, not being married later in life has been associated with suicidal ideation [88]. However, marital status (married, divorced, widowed, and never married) does not directly influence the HRV. The quality of marriage, understood as being continuously married/have remarried, and increases in marital satisfaction have been associated with higher HF-HRV [89]. Similarly, SNS reactivity (measured by pupil dilation while experiencing a stressor) was lower when participants were accompanied by their partners [90, 91]. This reduction was even more pronounced in function of the marital relationship quality [91]. Likewise, epinephrine levels were lower in older married participants than in non-married participants [92], and HRV was lower in women who lived alone than in those living in company [93].

Concerning *functional measures,* in their systematic review, Goodyke et al. [94] showed that perceived social support was positively associated with increased RMSSD and HF (two PNS activity measures) during rest, stress, and recovery after a stressor task. Other studies (not included in the review) also confirmed this association [93, 95]. Moreover, it has been proposed that HRV and social support are positively associated in conditions of high stress, and that this association is true for women but not men. This could suggest different coping strategies between sexes [96]. Moreover, in a sample of older participants, those with low self-reported social support displayed higher diastolic blood pressure [92].

Lastly, there is evidence supporting the hypothesis that not only receiving support but also giving support to others could reduce the SNS responses, which ultimately is beneficial for one’s health [97].

## From social Context to ANS Imbalance in Suicidal Patients

The results on the cardiovascular response, EDA and PLR suggest SNS hyperactivity and PNS hypoactivity, at rest and also in stress conditions, in suicidal patients. This indicates a constant ANS hyperactivation and thus an SNS/PNS imbalance in suicidal patients. Similarly, some of the nuclear social variables implicated in the theoretical framework of suicide are related to ANS imbalance. Indeed, distal variables (e.g. childhood maltreatment) are related to lower HRV. Moreover, proximal variables (e.g. SLE) are related to a SNS/PNS imbalance in resting conditions and in response to stress. The time interval between the SLE and the measurement is important when assessing ANS alterations; however, SLE accumulation would affect ANS via damage accumulation due to the allostatic load. Although the effect of loneliness and social isolation on the ANS response is less clear, previous studies consistently described a relationship between loneliness/isolation and vulnerability to cardiac disease [58]. These feelings, although devastating, are transitory and may act as moderators in ANS alteration. Conversely, structural and functional social support may act as moderators, increasing resilience and reducing the damage to stress systems.

**Fig. 1:**
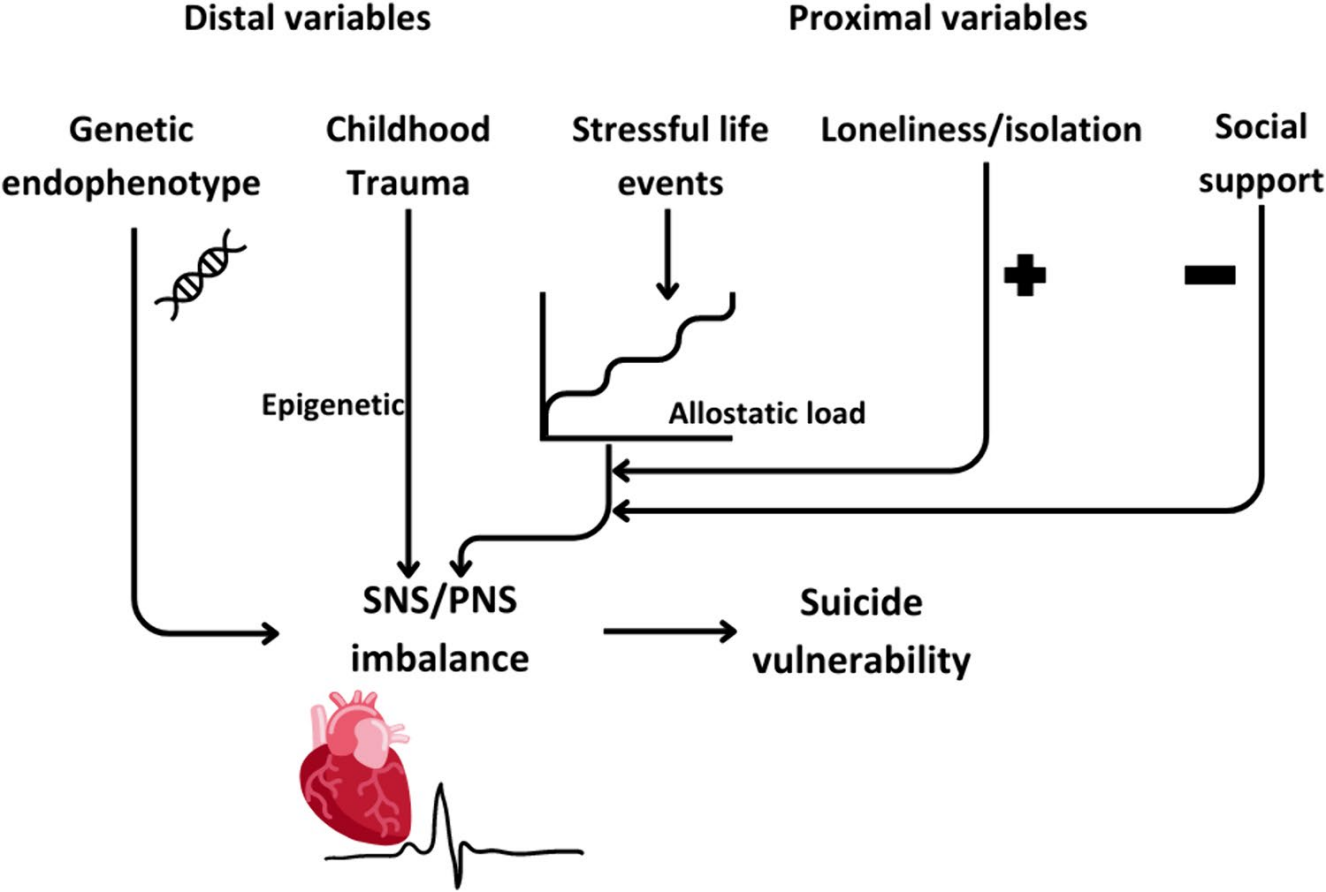
Mechanism of SNS/PNS impairment in suicidal patients

Thus, a SNS/PNS imbalance may be the result of genetic and epigenetic factors in conjunction with damage accumulation due to, in part, the social context (Fig. 1). In the neurovisceral integration model, PNS control is one of the most important control systems for the stress response [6]. Resting vagal tone is at the origin of the cognitive inhibitory control capacity via the prefrontal cortex [9]. Interestingly, vagal control via the prefrontal cortex is a heritable endophenotype. A reduced prefrontal cortex-mediated inhibitory control has been associated with lower HRV and also with impulsivity and suicidal behavior [98]. Another study showed that acute tryptophan depletion (to lower serotonin neurotransmission) decreased HRV and increased impulsivity, but only in individuals with history of suicidal ideation [99].

On the other hand, SNS/PNS imbalance could be explained by accumulated damage via epigenetic pathways or allostatic load. The inflammatory and endocrine systems might be implicated in ANS damage. The involvement of the inflammatory system in suicide has been demonstrated [100]. Interestingly, the PNS and particularly the vagal nerve (X) regulate inflammation: this is the "cholinergic anti-inflammatory pathway” [101]. Vagal afferents detect circulating pro-inflammatory cytokines in response to infection or injury and send the information to the central nervous system that starts a cholinergic response via the efferent vagal nerve. This inhibits the production of inflammatory cytokines. The results reported by Chang et al. [16] corroborated this anti-vagal inflammatory action: in individuals with depression, SI intensity was positively correlated with the serum concentration of high sensitivity C-reactive protein and negatively correlated with HF-HRV.

HPA axis is another signaling pathway that links suicidal risk and ANS. Dysregulated HPA axis response has been related to suicide [7, 102, 103]. The ANS and HPA axis are reciprocally innervated, interact, and are both controlled by the prefrontal cortex [104]. Cortisol liberated by the HPA axis maintains the effects of SNS activation [105] and increases the liberation of inflammatory factors, thus promoting neuroinflammation [106, 107]. Therefore, all three systems (ANS, HPA, and inflammation) are inter-correlated. Early life events and traumas may directly affect all stress systems via epigenetic mechanisms, thereby increasing the vulnerability to suicide [108]. SLE accumulation and chronic stress lead to the dysregulation of all three systems [109, 110].

## Conclusions

Several evidences support the hypothesis of a SNS/PNS imbalance in patients with suicidal spectrum behaviors. The ANS (SNS and SNP) is one of the key stress systems. Due to the importance of stress and social context in suicide, a non-adaptive stress response is a vulnerability factor for the whole suicidal spectrum. Notably, vagal control from PNS is a marker of better adaptability to social stress and social context and consequently, of better mental health. Indeed, PNS can downregulate other potentially harmful stress systems, such as the HPA axis and inflammation. It is not surprising that suicidal patients show a dysregulation in ANS systems, particularly in vagal control. Suicidal patients show difficulties in interpreting the social context, impairment in executive functions, and dysregulation of the HPA and inflammatory axes [5], all related to worse vagal control. Furthermore, social context distal and proximal variables partly explain ANS impairment, or act as moderators of the ANS response in patients in a stress condition.

Lastly, ANS alterations are not only useful as biomarkers or to explain suicidal ideation or behavior. They may also be useful for the specific treatment of suicidal ideation or suicide risk. For instance, these indicators can be used to assess the mechanisms of therapeutic change [22], and to develop applications for predicting the suicide risk [111]. Similarly, techniques, such as the respiratory-heart rate coherence biofeedback, can be used as specific treatments in patients with suicidal behavior [112].
